# Possible causes of divergent population trends in sympatric African herbivores

**DOI:** 10.1371/journal.pone.0213720

**Published:** 2019-03-12

**Authors:** Emily Bennitt, Tatjana Y. Hubel, Hattie L. A. Bartlam-Brooks, Alan M. Wilson

**Affiliations:** 1 Okavango Research Institute, University of Botswana, Maun, Botswana; 2 Structure and Motion Lab, Royal Veterinary College, London, United Kingdom; Sichuan University, CHINA

## Abstract

Sympatric herbivores experience similar environmental conditions but can vary in their population trends. Identifying factors causing these differences could assist conservation efforts aimed at maintaining fully functional ecosystems. From 1996–2013, tsessebe and wildebeest populations in the Okavango Delta, Botswana, declined by 73% and 90%, respectively, whereas zebra populations remained stable. These sympatric, medium sized herbivores are exposed to similar natural and anthropogenic pressures, but apparently differ in their responses to those pressures. To identify factors that could cause these differences, we fitted GPS-enabled collars to six zebra, eight tsessebe and seven wildebeest in the Moremi Game Reserve, Botswana. We calculated utilisation distributions (UDs) from GPS data, and used 95% isopleths to compare seasonal home range size between species. We calculated utilisation intensity (UI) from the UDs and generated spatial layers representing resources and disturbances, and then used model averaging to identify factors affecting UI for each species. We calculated second and third order habitat selection ratios to determine whether species were habitat specialists or generalists. Zebra occupied larger home ranges than tsessebe and wildebeest, showed weaker responses to spatial variables and displayed no third order habitat selection; zebra social systems are also more fluid, allowing for information exchange between stable harems. Herbivore species that are sedentary, occupy small home ranges, are habitat specialists and exist in relatively isolated groups are likely to be less resistant and resilient to the rapid pace of environmental change forecast by climate change scenarios. Resources contained within existing protected areas are unlikely to maintain populations of such species at sufficiently high levels, potentially leading to functional extinction. Special precautions may be needed to ensure that such species can persist in the wild, such as buffer zones around existing protected areas, which would allow greater potential for adaptive movement should current environmental conditions change.

## Introduction

Wildlife population trends fluctuate spatially and temporally with natural environmental variation, such that the same species in different environments can have divergent population growth patterns [1,2], as can sympatric species within one environment [3]. Globally, mammalian herbivore populations are declining in response to a variety of factors [4], mainly linked to anthropogenic impacts such as hunting [5], land-use changes [6], habitat loss and fragmentation [7], barriers to movement [8,9], and climate change [10]. These factors often create sudden environmental changes [11] that can affect behaviour [12], reproductive success [13] and gene flow between populations [14], ultimately impacting individual fitness and population dynamics [15].

Severe population declines can lead to functional extinction, whereby the ecological role played by a species can no longer be fulfilled by a small population [16], followed by local extinction, then species extinction under particular conditions [17]. Loss of ecological functionality can lead to trophic cascades, increasing the vulnerability of other species that rely on declining species, as seen in predator-prey relationships [18]. Species extinction risk is rarely random [19], but rather depends on a combination of life history traits and environmental variables [20]. Life history traits have evolved to maximise fitness and survival under current conditions [11], so changes to those conditions could lead to maladaptive behaviours and increased vulnerability [21,22].

Extinction risk is lowest in species with high capacity for movement [23], adaptive use of different and sometimes novel landscapes [12], broad geographic distributions [24] and low specificity of resource requirements [25]. Variation between species’ life history traits can affect their responses to changing environmental conditions within the same ecosystem [19]. Species vary in their resistance and resilience, which are their ability to withstand and recover from environmental change, respectively [19]. Usually species with low resistance also have low resilience; the latter is more important for population persistence [25]. All extant species have survived the rise of the Anthropocene [26], so they must be more resilient than many species that have already gone extinct [17], but their continued survival depends on their ability to adapt at a similar pace to that of predicted environmental change [27]. Herbivores are generally more vulnerable to population declines than carnivores because the latter can frequently switch food sources to domestic prey and are less specific in their habitat requirements, but herbivores are more numerous, and therefore population declines are not always as apparent [17].

Large herbivore populations in the Okavango Delta, Botswana, have been surveyed regularly for several decades by the Department of Wildlife and National Parks (DWNP), providing long term indicators of population trends. Between 1996 and 2013, populations of large bodied African elephant (*Loxodonta africana*) and Cape buffalo (*Syncerus caffer caffer*) remained relatively stable. Populations of some medium bodied herbivores, such as blue wildebeest (*Connochaetes taurinus*) and tsessebe (*Damaliscus lunatus lunatus*), declined by 73% and 90%, respectively (Chase 2010, unpublished report; Statistics Botswana 2014, unpublished report), while others, such as plains zebra (*Equus quagga*), remained stable. Population trends for sympatric herbivores with similar body sizes can therefore vary substantially, indicating different levels of resilience and resistance. To identify possible causes for this variation, we deployed GPS-enabled collars onto three medium sized species of African herbivore in the Okavango Delta: plains zebra, tsessebe and blue wildebeest. We combined GPS data from collars and geographical information systems (GIS) information to identify key factors affecting spatial utilisation. We quantified habitat selection and compared life history traits among the three species to identify potential causes of divergent population trends. We hypothesised that (i) zebra would have larger home ranges than tsessebe and wildebeest, (ii) tsessebe and wildebeest would be more sensitive to anthropogenic disturbances than zebra, and (iii) tsessebe and wildebeest would be more specialised in their habitat requirements than zebra. Our results provided some insight into the possible causes of divergent population trends in sympatric herbivores.

## Materials and methods

### Study area

**Fig 1:**
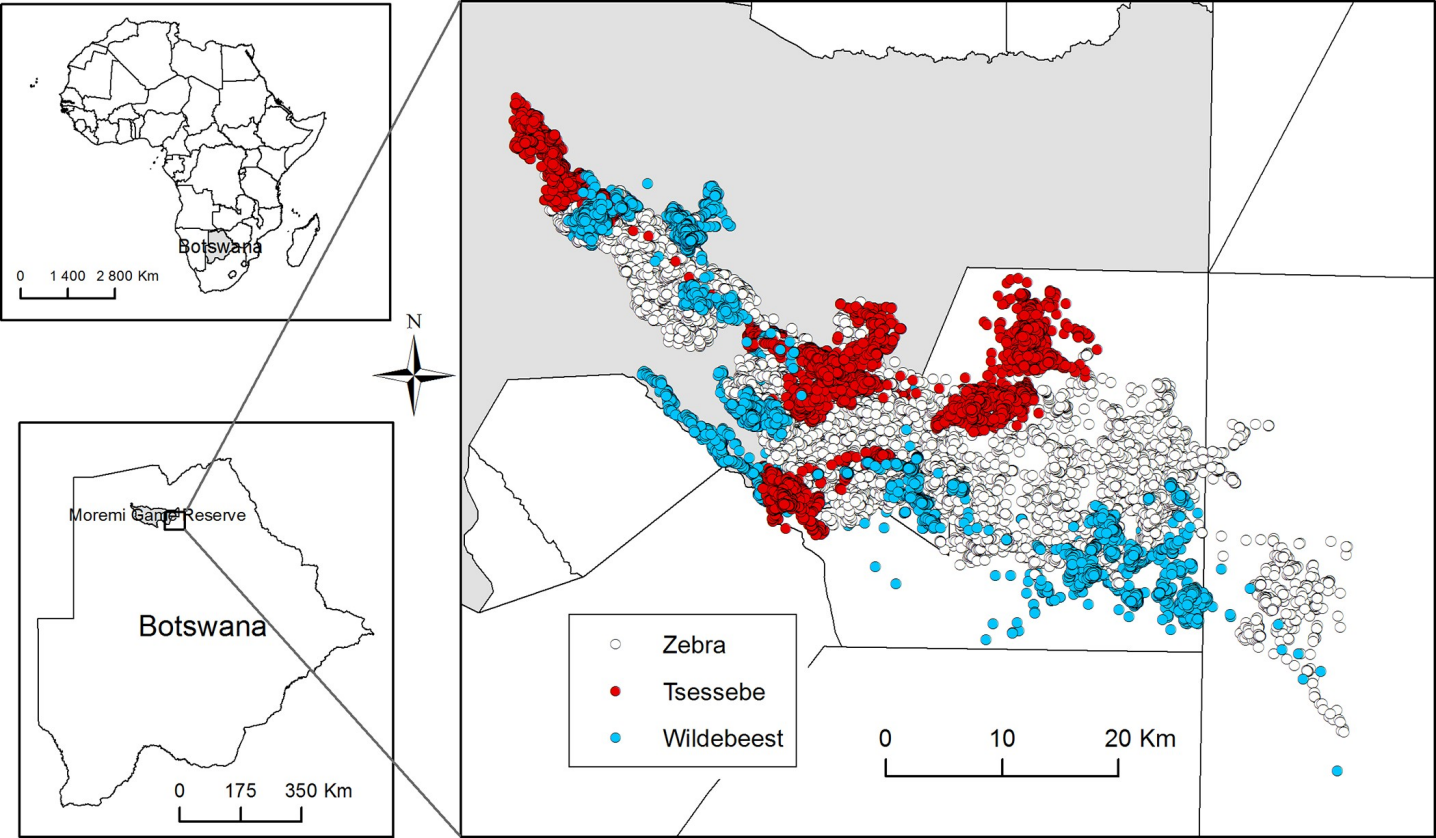
Map of study area detailing GPS locations of study species.

The Okavango Delta is located in northern Botswana, between 22.0° - 24.0° E and 18.5° - 20.5° S [28]. The region experiences two annual influxes of water, one from seasonal rains and the other from a delayed flooding response caused by rainfall in Angola [29]. Changing water levels caused by flooding and rainfall were used to define three seasons: the early flood season (April–July), when flood waters were rising; the late flood season (August–November), when flood waters were falling; and the rainy season (December–March), when most rainfall occurred. Our study area was in the south-eastern section of the Okavango Delta, including parts of Moremi Game Reserve and adjoining Wildlife Management Areas (Fig 1). The study species overlapped in their home ranges and movement paths (Fig 1), so had access to the same resources and were exposed to similar natural and anthropogenic pressures.

Six habitat types were defined based on differences in vegetation structure and type: grassland, riparian woodland, acacia woodland, mopane woodland, secondary floodplain and tertiary floodplain [30]. Secondary floodplain was flooded throughout the year, whereas tertiary floodplain flooded only during the peak flood. A habitat map was developed from georeferenced orthophotographs obtained from the Okavango Research Institute, Botswana, digitised at a 1:10,000 scale in ArcGIS 10.0 (ESRI, Redlands, California), then rasterised with a pixel size of 50 m. Ground-truthing points were used to calculate the accuracy of the map at 88.1% [30].

### Collaring

Six zebra, eight tsessebe and seven wildebeest were fitted with custom-built GPS-enabled collars programmed to record GPS fixes every five minutes, developed by the Structure and Motion Laboratory of the Royal Veterinary College, London [31–33]. Females were collared because they represented core breeding herd members, and because males were more likely to cause damage to collars through fighting. All animals belonged to separate groups. Zebra were darted in November 2014 and fitted with collars that included remote drop-off units (Sirtrack, Hawkes Bay, New Zealand), programmed to trigger in October 2015, removing the need to immobilise animals for collar recovery. All tsessebe and five wildebeest were darted in September 2015, and two wildebeest were darted in January 2016. All tsessebe and wildebeest collars were programmed to drop off in October 2016. Collars deployed on two zebra, three tsessebe and four wildebeest suffered electronic failure before the year elapsed, one tsessebe collar suffered structural failure and one wildebeest collar was recovered following a suspected predation event. All drop-offs on zebra collars functioned as programmed, whereas three tsessebe and three wildebeest had to be darted to recover collars following drop-off failure. All collars with failures were recovered and examined to improve future generations of collars. Zebra, tsessebe and wildebeest were collared for mean ± S.D. of 266 ± 59 days, 327 ± 156 days, and 250 ± 146 days, respectively.

All tsessebe, all zebra and five wildebeest were darted from vehicles, and two wildebeest were darted from a helicopter. Zebra were immobilised with 7 mg Etorphine, 80 mg Azaperone, and 1667 i.u. Hyalase. Tsessebe were immobilised with 4 mg Thiafentanil, 80 mg Azaperone, 1667 i.u. Hyalaze, and topped up post capture with 100 mg ketamine. Wildebeest were immobilised with 5 mg Etorphine, 80 mg Azaperone, and 1667 i.u. Hyalase. All animals were reversed with 50 mg Naltrexone. Dart sites were injected with a systemic analgesic and local antibiotics to prevent infection. Heavily pregnant females and those with small young were avoided. All animals were observed rejoining their original groups following darting, with no ill effects. All dartings were carried out by a qualified veterinarian experienced with game capture and registered in Botswana. Prior to darting, permits were secured from the DWNP based on research permit EWT 8/36/4 XXIV (199). All animal handling procedures followed the guidelines from the American Society of Mammalogists [34] and were carried out under ethical approval from the Ethics and Welfare Committee of the Royal Veterinary College, London (URN 2013 1233). Fieldwork was conducted on government-owned, protected land that included a Game Reserve and several Wildlife Management Areas. No endangered or protected species were sampled.

### Utilisation distributions

We calculated seasonal utilisation distributions (UDs) for each individual using the movement-based kernel density estimator (MKDE) from the “adehabitat” package in R v.3.3.2 (R Core Development Team, 2017). The MKDE takes the temporal sequence of fixes into account, as well as movement rates across different habitat types [35,36]. The MKDE requires a minimum distance threshold (MDT), below which animals are considered to be resting [36]. Short distances between fixes taken at five minute intervals could indicate resting or intensive grazing, which are very different levels of habitat use. We therefore downsampled the data to hourly fixes and used 50 m as the MDT, the same value as the spatial layer pixel size. UDs represent relative utilisation intensity (UI), so can be generated from unbalanced datasets [37], including from animals that were not collared for entire seasons, although we removed data from animals collared for less than two months in a given season. We calculated seasonal home range size from the 95% UD isopleths of animals that were collared for at least three months in a given season. We ran linear mixed models on the log of home range size to determine the effects of species and season after running a Shapiro-Wilk test for normality; individual was included as a random effect. We identified the most parsimonious models using Akaike’s Information Criterion (AIC) [38].

### Spatial layers

Secondary floodplain was flooded throughout the year, so was identified as a permanent water source and used to generate a spatial layer with pixel values for distance to permanent water (2FP). Every ephemeral pan in the study area was identified and mapped using Google Earth (Mountainview, CA, USA). Pans have a distinctive darker soil colour and are located within gaps in wooded canopy, so could be readily identified regardless of the time of year of the Google Earth image [39]. Pan locations were used to generate a pan density layer, with each pixel having a value for the number of pans within a 50 m radius, corresponding to the pixel size (Pans). Spatial data from the Okavango Delta Management Plan (DWNP, unpublished report, 2008) were used to map anthropogenic features such as veterinary fences, villages, and lodges, which have not changed location in the intervening years. Layers were generated using the distance to fences (Fence) and the distance to human settlements, villages and lodges (Villodge). Tracks4Africa (Johannesburg, South Africa) provided shapefiles of roads in Moremi Game Reserve, which we combined with our own databases from surrounding areas to generate a layer based on the distance to roads (Roads). We downloaded Normalised Difference Vegetation Index data from MODIS (NDVI) and rainfall data from PERSIANN (Rain) and converted them from 0.25 decimal degrees to 50 m pixel resolution. This allowed data analysis, but did not provide any more detailed information about NDVI and rainfall than the original resolution.

We extracted data values from each spatial layer for every seasonal UD, using the 95% isopleths as bounding polygons. The relatively small pixel size of the spatial layers generated a large amount of data, so we randomly extracted values from 5 000 pixels per UD without replacement for analysis. We ran generalised linear mixed models with Gaussian correlation structures based on GPS coordinates to account for spatial autocorrelation using the “nlme” package in R [40]. UI was the dependent variable; 2FP, Pans, Fence, Villodge, Roads, NDVI and Rain were the fixed effects; and individual was the random effect. We standardized the parameter values to remove any bias. We used the “dredge” function from the “MuMIn” package in R to identify all models with ΔAIC<2, then used the “model.avg” function from the same package to identify variables to be included in the best model and to estimate model averaged parameter values [41].

### Habitat selection

We generated population level seasonal Minimum Convex Polygons (MCPs) from all individuals in each species, and compared proportion of habitat used within the 95% UD isopleths to proportion of habitat available in the population level MCPs as second order habitat selection, which represents the home range selected by an individual or group [42]. We calculated proportional habitat use weighted by intensity from the UDs and compared it to proportional habitat availability within the 95% UD isopleths as third order habitat selection, which represents how animals use resources within their home ranges [42]. We calculated second and third order seasonal Manly selection ratios for each species, whereby selection ratios with 95% confidence intervals >1 and <1 represent selection and avoidance, respectively [43]. For second and third order selection, we ran MANOVAs on the selection ratios with the fixed effects of season and species, and the interaction between the two. We used AIC values to select the most parsimonious models.

## Results

Not all animals were collared for complete seasons (Table 1), and animals collared for less than a month within a season were discarded from analyses, so sample sizes varied with analysis methods.

**Table 1:** Duration of collared period for zebra, tsessebe and wildebeest, marked as shaded.

	Late flood			Rainy				Early flood				Late flood			
Individual	S	O	N	D	J	F	M	A	M	J	J	A	S	O	N
Zebra 1															
Zebra 2															
Zebra 3															
Zebra 4															
Zebra 5															
Zebra 6															
Tsessebe 1															
Tsessebe 2															
Tsessebe 3															
Tsessebe 4															
Tsessebe 5															
Tsessebe 6															
Tsessebe 7															
Tsessebe 8															
Wildebeest 1															
Wildebeest 2															
Wildebeest 3															
Wildebeest 4															
Wildebeest 5															
Wildebeest 6															
Wildebeest 7															

Zebra were first collared in late 2014; tsessebe and wildebeest were first collared in late 2015 or early 2016.

### Seasonal home ranges

**Fig 2:**
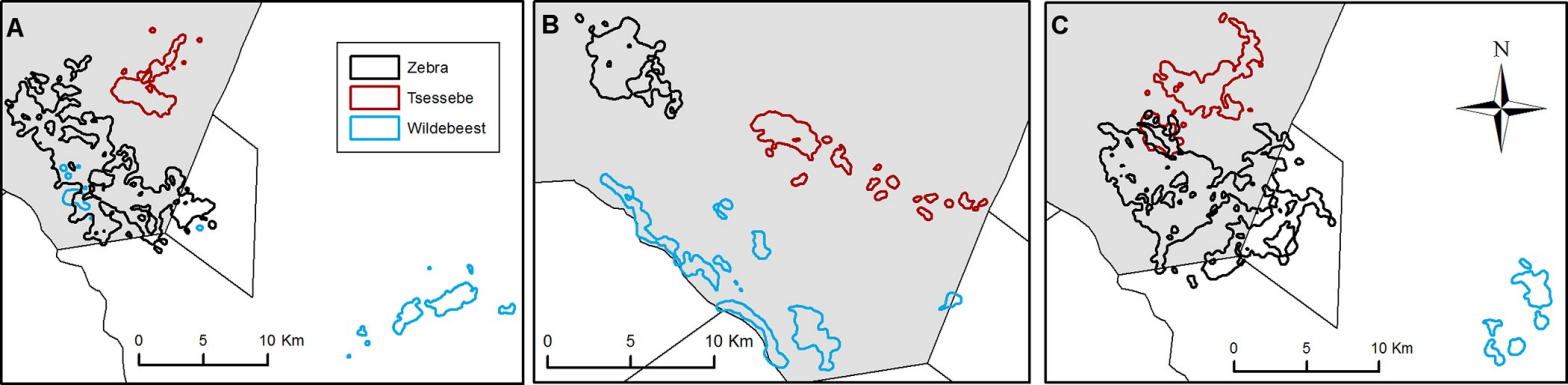
**Examples of (A) early flood, (B) late flood, and (C) rainy season home ranges for zebra (Zebra 2), tsessebe (Tsessebe 5) and wildebeest (Wildebeest 1) occurring in similar locations within the study area**.

**Fig 3:**
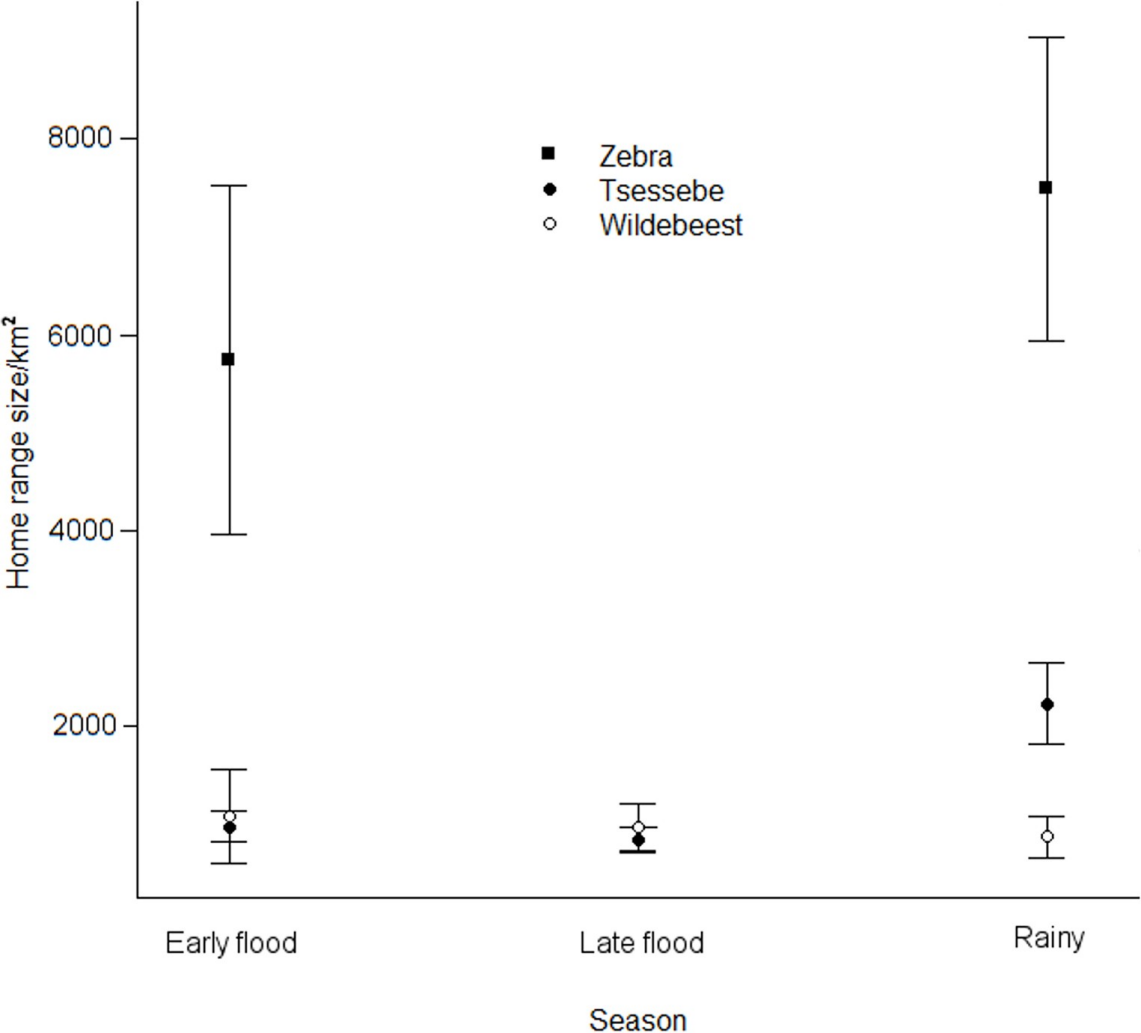
Seasonal home range sizes of zebra, tsessebe and wildebeest in the Okavango Delta, Botswana. Error bars represent S.E.

To compare seasonal home range sizes, we used data from animals that were collared for at least three months out of each season, during which time they were assumed to cover their full seasonal home range. No data were included from zebra during the late flood season because we only had data from animals that were collared for half the season, which could have affected their patterns of space use. Analyses were run with data from five zebra, six tsessebe and four wildebeest during the early flood season, from seven tsessebe and five wildebeest during the late flood season and from six zebra, six tsessebe and five wildebeest during the rainy season (e.g. Fig 2). The most parsimonious model included the fixed effects of season and species (AIC = 92.34, AIC_ω_ = 0.51), but the model with only the fixed effect of species was also competitive (ΔAIC = 0.07, AIC_ω_ = 0.49), indicating that the effect of species was slightly more important than the effect of season. Zebra had larger home ranges than the other two species, which only differed during the rainy season, when tsessebe home ranges were larger than those of wildebeest (Fig 3). Zebra and tsessebe home ranges were largest in the rainy season (Fig 3).

### Spatial models

For spatial modelling, we used data from animals that were collared for at least two months out of every season because the UDs allowed for the inclusion of partial seasonal datasets. Analyses were run with data from five zebra, six tsessebe and five wildebeest during the early flood season, from four zebra, eight tsessebe and six wildebeest during the late flood season, and from six zebra, six tsessebe and five wildebeest during the rainy season. For each species and season, we identified between two and four competitive models, which we averaged to produce the best parameter estimates for variables with an effect on UI (Table 2). Variables affecting UI differed with season and species, and distance to permanent water was the only variable present in every model. In all seasons, zebra parameter values were lower than the other two species, suggesting that the effects of the parameters on zebra UI were not as substantial as on tsessebe and wildebeest UI (Table 2). During the early flood season, pan density was the only factor that increased UI for every species (Table 2). Zebra and tsessebe used areas closer to permanent water and fences, in contrast to wildebeest that avoided those areas. During the late flood and rainy seasons, zebra and wildebeest utilised areas closer to permanent water, whereas tsessebe preferred locations further from permanent water (Table 2). During the late flood season, tsessebe and wildebeest UI was higher closer to roads, whereas zebra UI was higher further from roads (Table 2). During the rainy season, high levels of NDVI were linked with low levels of UI for all species; high rainfall was also linked to low UI for tsessebe and wildebeest (Table 2).

**Table 2:** Model averaged parameter values explaining seasonal variation in spatial utilisation intensity for herbivores in the Okavango Delta, Botswana.

**Season**	**Species**	**Parameter**	**Estimate**	**Unconditional standard error**	**Confidence intervals**	**Relative importance**
Early flood	Zebra	**2FP**	**-1.47**	**0.49**	**-2.42, -0.52**	**1.00**
		**Fence**	**-2.40**	**0.64**	**-3.65, -1.14**	**1.00**
		NDVI	N/A	N/A	N/A	0.00
		**Pans**	**1.35**	**0.29**	**0.78, 1.91**	**1.00**
		Roads	0.46	0.27	-0.08, 1.00	0.60
		**Villodge**	**2.27**	**0.37**	**1.53, 2.99**	**1.00**
	Tsessebe	**2FP**	**-41.84**	**5.59**	**-52.79, -30.88**	**1.00**
		**Fence**	**-122.0**	**11.64**	**-142.83, 397.17**	**1.00**
		NDVI	-0.63	1.71	-6.84, 3.08	0.33
		**Pans**	**23.28**	**4.60**	**14.27, 32.30**	**1.00**
		**Roads**	**20.42**	**1.74**	**17.01, 23.82**	**1.00**
		Villodge	-1.29	1.99	-6.85, 1.34	0.47
	Wildebeest	**2FP**	**13.44**	**6.38**	**0.94, 25.94**	**1.00**
		**Fence**	**115.40**	**21.22**	**73.84, 157.04**	**1.00**
		**NDVI**	**15.46**	**3.99**	**7.65, 23.28**	**1.00**
		**Pans**	**17.12**	**3.96**	**9.36, 24.89**	**1.00**
		Roads	-0.07	2.12	-8.78, 4.53	0.31
		**Villodge**	**-42.10**	**7.51**	**-56.82, -27.38**	**1.00**
Late flood	Zebra	**2FP**	**-5.47**	**0.95**	**-7.32, -3.61**	**1.00**
		Fence	-0.16	0.70	-3.50, 1.94	0.20
		**NDVI**	**6.01**	**0.83**	**4.37, 7.64**	**1.00**
		**Pans**	**13.15**	**1.39**	**10.42, 15.87**	**1.00**
		**Roads**	**6.94**	**0.88**	**5.22, 8.67**	**1.00**
		Villodge	-0.16	1.36	-3.50, 1.94	0.67
	Tsessebe	**2FP**	**39.23**	**6.22**	**26.45, 50.67**	**1.00**
		**Fence**	**151.0**	**23.45**	**102.29, 188.09**	**1.00**
		NDVI	3.54	3.23	-0.05, 10.3	0.69
		**Pans**	**-17.78**	**6.59**	**-30.86, -5.05**	**1.00**
		**Roads**	**-15.47**	**2.42**	**-20.17, -10.69**	**1.00**
		**Villodge**	**20.30**	**4.64**	**10.98, 29.14**	**1.00**
	Wildebeest	**2FP**	**-18.11**	**4.17**	**-26.28, -9.94**	**1.00**
		Fence	-0.53	4.12	-24.07, 16.65	0.14
		NDVI	-5.32	4.08	-13.45, 0.24	0.81
		Pans	-3.93	5.29	-17.67, 2.12	0.51
		**Roads**	**-26.01**	**3.81**	**-33.48, -18.55**	**1.00**
		**Villodge**	**-10.49**	**4.74**	**-19.78, -1.20**	**1.00**
Rainy	Zebra	**2FP**	**-1.26**	**0.52**	**-2.28, -0.24**	**1.00**
		**Fence**	**-2.36**	**0.65**	**-3.64, -1.08**	**1.00**
		**NDVI**	**-1.00**	**0.33**	**-1.64, -0.36**	**1.00**
		Pans	-0.06	0.26	-0.56, 0.44	0.21
		Rain	-0.16	0.62	-1.37, 1.04	0.22
		**Roads**	**-0.96**	**0.23**	**-1.42, -0.50**	**1.00**
		**Villodge**	**0.77**	**0.33**	**0.19, 1.42**	**1.00**
	Tsessebe	**2FP**	**11.40**	**2.06**	**7.36, 15.44**	**1.00**
		Fence	-1.55	2.55	-9.00, 2.02	0.44
		**NDVI**	**-16.59**	**1.50**	**-19.53, -13.65**	**1.00**
		**Pans**	**-14.70**	**2.22**	**-19.05, -10.35**	**1.00**
		**Rain**	**-23.45**	**2.02**	**-27.41, -19.49**	**1.00**
		**Roads**	**9.24**	**1.12**	**7.04, 11.44**	**1.00**
		Villodge	1.62	1.74	-1.79, 5.02	0.37
	Wildebeest	**2FP**	**-56.17**	**11.27**	**-78.26, -34.09**	**1.00**
		Fence	-0.69	5.69	-26.61, 20.21	0.21
		**NDVI**	**-35.53**	**4.35**	**-44.06, -27.00**	**1.00**
		Pans	6.08	4.77	-0.03, 15.60	0.78
		**Rain**	**-7.89**	**3.47**	**-14.69, -1.09**	**1.00**
		**Roads**	**-13.02**	**2.46**	**-17.84, -8.20**	**1.00**
		**Villodge**	**-3.41**	**8.87**	**-51.49, -16.72**	**1.00**

All values are e^-9^ except for Relative importance. Significant parameters are highlighted in bold. Abbreviations: 2FP = Distance to permanent water; Fence = Distance to fence; NDVI = Normalised Difference Vegetation Index; Pans = Density of ephemeral waterholes within 50 m radius; Rain = Rainfall; Roads = Distance to roads; Villodge = Distance to village or lodge

### Habitat selection

We used the same dataset for habitat selection analyses as for spatial modelling.

#### Second order habitat selection

During the early flood season, second order habitat selection was significant for zebra (X^2^_5_ = 302.07, p<0.001), tsessebe (X^2^_5_ = 87.39, p<0.001), and wildebeest (X^2^_5_ = 120.308, p<0.001). All species avoided secondary floodplain, and zebra avoided mopane woodland and selected grassland, but the other species showed no preference for any other habitats (Table 3). During the late flood season, second order habitat selection was significant for zebra (X^2^_5_ = 208.72, p<0.001), tsessebe (X^2^_5_ = 575.53, p<0.001), and wildebeest (X^2^_5_ = 308.47, p<0.001). All species avoided mopane woodland (Table 3). Zebra and tsessebe selected grassland, and zebra and wildebeest avoided acacia woodland, which was not present in any of the wildebeest home ranges (Table 3). Tsessebe and wildebeest selected tertiary floodplain, and tsessebe avoided secondary floodplain (Table 3). During the rainy season, second order habitat selection was significant for zebra (X^2^_5_ = 422.65, p<0.001), tsessebe (X^2^_5_ = 323.55, p<0.001), and wildebeest (X^2^_5_ = 33.67, p<0.001). Zebra and tsessebe avoided secondary floodplain and selected grassland (Table 3). Zebra also avoided mopane woodland and selected riparian woodland (Table 3). Wildebeest did not display selection or avoidance of any particular habitats (Table 3).

**Table 3:** Habitat selection ratios for herbivore species in the Okavango Delta, Botswana.

**Season**	**Habitat**	**Selection order**	**Habitat selection ratios (95% confidence intervals)**		
			Zebra	Tsessebe	Wildebeest
Early flood	Acacia woodland	Second	0.33 (-0.43–1.09)	1.19 (-0.55–2.93)	0.78 (-0.33–1.89)
		Third	0.97 (0.88–1.06)	0.94 (0.72–1.16)	0.71 (0.32–1.09)
	Secondary floodplain	Second	**0.43 (0.02–0.66)**	**0.17 (-0.18–0.52)**	**0.37 (-0.15–0.89)**
		Third	1.05 (-0.10–2.20)	1.38 (0.69–2.06)	1.52 (0.26–2.78)
	Tertiary floodplain	Second	1.19 (0.48–1.90)	0.66 (-0.63–1.94)	2.24 (-0.15–4.64)
		Third	1.14 (0.40–1.88)	1.06 (0.41–1.71)	**1.55 (1.09–2.00)**
	Mopane woodland	Second	**0.34 (0.02–0.66)**	0.94 (-0.03–1.91)	0.51 (-0.11–1.13)
		Third	**0.68 (0.44–0.92)**	0.81 (0.34–1.28)	0.99 (0.35–1.64)
	Grassland	Second	**2.01 (1.48–2.53)**	1.38 (0.57–2.20)	1.53 (0.51–2.55)
		Third	**1.13 (1.00–1.27)**	1.20 (0.91–1.49)	1.05 (0.82–1.29)
	Riparian woodland	Second	1.78 (1.32–2.24)	0.98 (-0.45–2.41)	0.98 (0.24–1.71)
		Third	**0.78 (0.65–0.91)**	0.85 (0.49–1.22)	**0.67 (0.42–0.92)**
Late flood	Acacia woodland	Second	**0.16 (-0.26–0.58)**	0.42 (-0.68–1.52)	**0.00 (0.00–0.00)**
		Third	**0.56 (0.56–0.56)**	0.84 (0.83–0.85)	N/A
	Secondary floodplain	Second	1.04 (-0.10–2.18)	**0.38 (-0.05–0.81)**	1.18 (0.53–1.83)
		Third	0.80 (0.53–1.07)	**0.49 (0.26–0.71)**	1.09 (0.57–1.62)
	Tertiary floodplain	Second	1.74 (0.64–2.84)	**2.63 (1.19–4.07)**	**2.68 (1.70–3.66)**
		Third	1.47 (0.96–1.98)	**1.33 (1.11–1.54)**	1.15 (0.77–1.53)
	Mopane woodland	Second	**0.19 (-0.15–0.52)**	**0.05 (-0.04–0.15)**	**0.12 (-0.09–0.34)**
		Third	**0.58 (0.50–0.66)**	**0.32 (0.22–0.41)**	**0.41 (0.33–0.49)**
	Grassland	Second	**1.48 (1.15–1.81)**	**1.55 (1.20–1.89)**	0.93 (0.42–1.44)
		Third	1.03 (0.83–1.23)	0.94 (0.76–1.12)	1.03 (0.69–1.37)
	Riparian woodland	Second	1.59 (0.99–2.20)	1.05 (0.57–1.52)	0.92 (0.62–1.22)
		Third	0.91 (0.75–1.06)	0.97 (0.81–1.14)	**0.66 (0.58–0.75)**
Rainy	Acacia woodland	Second	0.47 (-0.28–1.21)	0.45 (-0.17–1.07)	0.72 (-0.15–1.59)
		Third	**1.20 (1.00–1.39)**	1.62 (0.21–3.02)	0.84 (0.55–1.14)
	Secondary floodplain	Second	**0.50 (0.00–0.99)**	**0.16 (-0.15–0.47)**	2.02 (-2.09–6.14)
		Third	1.15 (0.73–1.58)	**0.47 (0.19–0.76)**	1.40 (0.42–2.39)
	Tertiary floodplain	Second	1.16 (0.07–2.26)	**0.41 (-0.41–1.24)**	1.59 (-1.33–4.51)
		Third	0.83 (0.54–1.13)	**0.25 (0.12–0.38)**	1.06 (0.14–1.97)
	Mopane woodland	Second	**0.35 (0.10–0.61)**	0.50 (-0.15–1.14)	0.88 (-0.43–2.18)
		Third	0.79 (0.42–1.17)	**0.59 (0.26–0.92)**	1.00 (0.93–1.06)
	Grassland	Second	**2.42 (1.70–3.14)**	**2.11 (1.29–2.94)**	1.15 (0.11–2.19)
		Third	**1.06 (1.02–1.09)**	**1.20 (1.07–1.34)**	1.15 (0.58–1.72)
	Riparian woodland	Second	**2.20 (1.17–3.24)**	1.39(0.18–2.63)	1.18 (-0.11–2.48)
		Third	0.89 (0.71–1.07)	0.87 (0.67–1.08)	**0.59 (0.39–0.79)**

Significant results are in bold.

**Fig 4:**
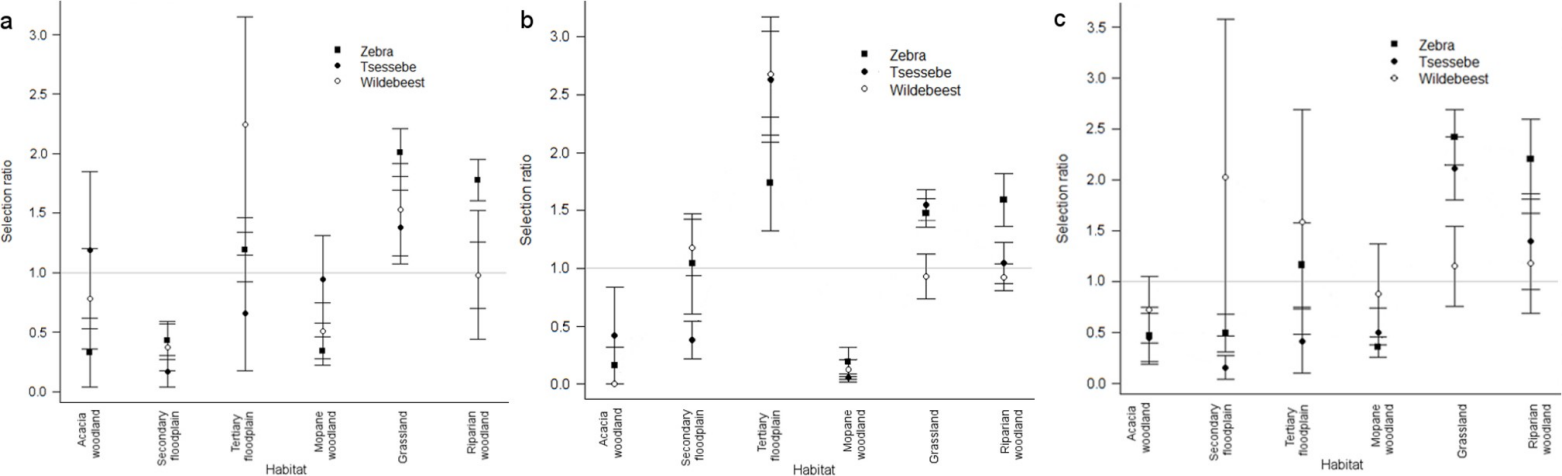
**Second order habitat selection ratios for zebra, tsessebe and wildebeest in the Okavango Delta, Botswana, during the (a) early flood, (b) late flood and (c) rainy seasons.** Error bars represent S.E.

When comparing selection ratios, the most parsimonious model included the interaction effect of species and season (AIC = 450.23). Floodplain habitats had higher selection ratios in the early and late flood seasons, and lower selection ratios in the rainy season, although wildebeest selected tertiary floodplain in the rainy season and tsessebe avoided tertiary floodplain during the early flood season (Fig 4). Zebra and tsessebe had higher selection ratios for grassland than wildebeest during the late flood and rainy seasons, and zebra showed a stronger preference for riparian woodland than other species in all seasons (Fig 4).

#### Third order habitat selection

During the early flood season, third order habitat selection was not significant for zebra (X^2^_23_ = 16.54, p = 0.831), but was significant for tsessebe (X^2^_21_ = 44.63, p = 0.002) and wildebeest (X^2^_19_ = 34.43, p = 0.016). Tsessebe did not show selection or avoidance of any particular habitats, whereas wildebeest selected tertiary floodplain and avoided riparian woodland (Table 3). During the late flood season, neither zebra (X^2^_16_ = 12.77, p = 0.689) nor tsessebe (X^2^_27_ = 32.94, p = 0.199) displayed third order habitat selection, but wildebeest did (X^2^_24_ = 54.10, p<0.001). Wildebeest did not select any particular habitats, but they avoided mopane and riparian woodlands (Table 3). No wildebeest home ranges included acacia woodland, so there were no third order selection ratios available for that habitat type. During the rainy season, zebra did not display any third order habitat selection (X^2^_25_ = 14.05, p = 0.961), but tsessebe (X^2^_21_ = 41.63, p = 0.005) and wildebeest did (X^2^_16_ = 32.69, p = 0.008). Tsessebe selected for grassland and avoided both floodplain types and mopane woodland, whereas wildebeest did not select any particular habitats but avoided riparian woodland (Table 3).

**Fig 5:**
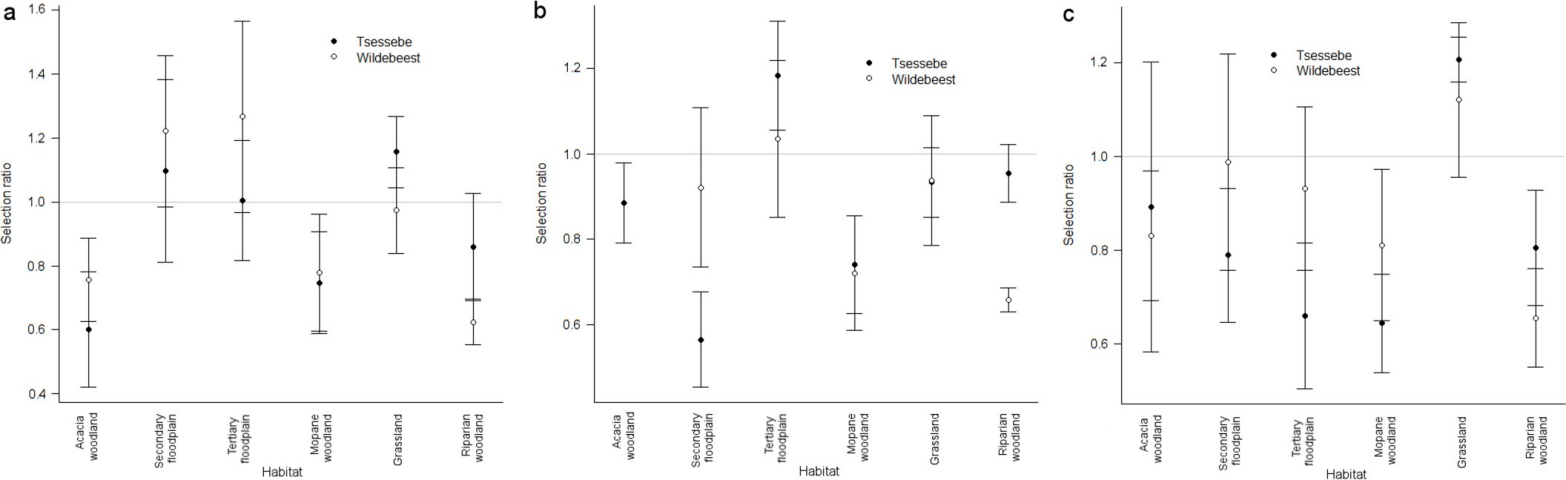
**Third order habitat selection ratios for zebra, tsessebe and wildebeest in the Okavango Delta, Botswana, during the (a) early flood, (b) late flood and (c) rainy seasons**. Error bars represent S.E.

Zebra did not display any third order habitat selection, so were removed from the MANOVA analysis to compare selection ratios between species and seasons. The most parsimonious model included the interaction effect of species and season (AIC = -83.22, AIC_ω_ = 0.69), but the model with fixed effects of season and species was also competitive (ΔAIC = 1.58, AIC_ω_ = 0.31). In all seasons, tsessebe showed a stronger preference for riparian woodland than wildebeest, whereas wildebeest showed a stronger preference for secondary floodplain than tsessebe (Fig 5).

## Discussion

Sympatric herbivores have access to similar resources and are adapted to co-exist within their environment [44]. Zebra, tsessebe and wildebeest have similar body masses, but differ in their life history traits, with different digestive systems, social organisation, mouth morphology, and resource requirements. In the Okavango Delta, zebra home ranges were larger than those of tsessebe and wildebeest, and the first two species had larger home ranges during the rainy season. None of the species showed particular avoidance for indicators of anthropogenic activity, such as proximity to roads or villages, and water availability seemed to be the main driver of herbivore distribution. All species displayed second order habitat selection, but, whereas tsessebe and wildebeest also displayed third order habitat selection, zebra used the habitats within their home ranges in proportion to their availability. Wildebeest showed a stronger preference for floodplain habitats than the other two species. When selection was significant, most woodland habitats were avoided by all species, although zebra showed some preference for riparian woodland, especially during the rainy season.

We were not able to include all possible factors that could affect distribution, such as competition or predation risk, because we could not collar every herd or predator within the study area. This was a short-term study because of financial and logistical constraints, and so could not yield sufficient information to exactly explain long-term population trends, but rather offered an insight into possible explanations that need further investigation. Small scale studies rarely provide information that can be applied at a broad scale, but are more likely to produce accurate results that can be used rapidly by local environmental managers [45]. Data were obtained from zebra one year prior to data obtained from tsessebe and wildebeest, so varying environmental conditions could have contribute to observed differences. Rainfall in 2014/2015 was fairly low, totalling 305.7 mm compared to 429.7 mm in 2015/2016 (http://okavangodata.ub.bw/ori/monitoring/rainfall/, accessed 20/02/2019). However, maximum flood extent in 2015 was 8883.5 km^2^, higher than the 7272.75 km^2^ flooded in 2016 (http://okavangodata.ub.bw/ori/monitoring/flood_maps/, accessed 20/02/2019). Higher flood levels could have reduced the amount of space available to herbivores, thereby causing them to occupy smaller home ranges, but the opposite trend was observed, so it seems unlikely that flooding extent would have affected home range size.

Zebra and tsessebe home ranges were largest during the rainy season, when abundant forage and water would have allowed dispersal across the landscape, enabling tsessebe to occupy rainy season home ranges that were twice the size of home ranges in the other seasons. Wildebeest home range size remained constant throughout the year, indicating that the productive rainy season did not increase their ranging capabilities, possibly because the short grasses that they favour were restricted in their distribution. Zebra occupied larger home ranges, which would encompass a broader range of habitats and resources than those of tsessebe and wildebeest. Zebra are therefore more vagile and less restricted in their movements, so they should be better able to compensate for environmental changes [46]. All collared animals were part of resident populations, living in the Moremi Game Reserve and surroundings all year, but zebra elsewhere in northern Botswana perform migrations [47], including the longest in Africa [48]. Wildebeest in East Africa are highly mobile and have large population sizes [49], but historically large wildebeest migrations in Botswana have mostly died out [50], with the exception of the Makgadikgadi Pans, where their numbers are far below those of zebra (Statistics Botswana 2014, unpublished report). Wildebeest in Botswana therefore appear to be highly sensitive to disturbances that restrict their movements, including fences and artificial water provision [50].

Small home ranges could lead to higher predation risk from predators looking for a reliable food source [51]. Wildebeest and zebra are favoured by lion (*Panthera leo*), whereas the high speeds and manoeuvrability of tsessebe make them a better target for African wild dog (*Lycaon pictus*) or cheetah (*Acinonyx jubatus*) (Hubel et al., unpublished data). Wild dog tend to run until they encounter prey opportunistically [32], whereas lion will target prey, often at night, that is easy to catch [52]. Wildebeest in the Central Kalahari Game Reserve, Botswana, rarely move far from artificial water sources, and are frequently predated by lion hunting predictable prey [50].

All collared animals were females and therefore represented breeding herds, each usually associated with only one adult male, several adult females and their associated offspring. Such breeding herds are most likely to behave in relatively similar ways across species, so provided a better frame of reference than a comparison between, for example, animals of different genders. Zebra and tsessebe occur in small, stable harems [53], but zebra move freely across their home range, whereas tsessebe harems often hold territories [54]. Within our study area, tsessebe rarely joined up with other harems, whereas zebra regularly did, and could form herds of several thousand individuals, as in the Makgadikgadi Pans, Botswana [47]. Wildebeest occur in breeding herds of several dozen animals that join a territory-holding male, although they can move outside of that territory during nomadic phases [55]. These social systems reflect varying levels of behavioural flexibility and could be linked to information exchange about resource availability. Nomadic zebra could move freely and track changing resource availability in natural landscapes [56], in contrast to territorial tsessebe and wildebeest. Zebra may therefore be better able to adapt to the changing environmental conditions and spatio-temporal distribution of resources predicted by climate change scenarios [46].

Distance to permanent water was the main spatial variable determining herbivore distribution during the flood seasons. Zebra showed a relatively weak preference for areas close to water during both seasons, whereas tsessebe preferred areas close to water during the early flood season and areas away from water during the late flood season, and wildebeest had opposing selection patterns to tsessebe. Avoidance of permanent water could have been linked to predation risk, since predators often visit water sources, and can influence herbivore behaviour [57]. Species avoiding permanent water may have been foraging in areas further from floodplains as an anti-predator strategy, although they would have needed to visit permanent water sources to drink, particularly during the late dry season. Most climate change scenarios predict rising temperatures, which could lead to stronger water dependence and restrict herbivore movement in relation to water [16], potentially increasing predation risk.

During the rainy season, all species avoided areas with high NDVI, and tsessebe and wildebeest avoided areas with high rainfall. High NDVI levels are associated with highly productive grass growth [58], but the resolution of NDVI and rainfall data was substantially lower than that of the other variables, which could have affected the results. Alternatively, avoidance of high NDVI and rainfall areas could be linked to grass height. Tsessebe and wildebeest, with their relatively narrow mouths, are more specialised feeders than zebra [59]. Herbivore species with narrow mouths favour short grass areas, where they can feed more selectively [60], so tsessebe and wildebeest may have been avoiding tall grass areas associated with high rainfall.

Spatial features related to anthropogenic activity did not appear to influence species distribution. Human activity is increasing in the Okavango Delta and surroundings [61], but it still has a relatively low impact. Tourists are not allowed to leave roads in the Moremi Game Reserve, nor can they drive at night. Structurally, unpaved roads used by tourists are similar to large game trails, and are used as such by many species [62]. The study area was relatively far from fences and only included a handful of lodges, so their impact was minimal [9]. Road networks were developed by park officials to maximise the chances of tourists encountering wildlife in productive habitats, so this could explain why herbivores were sometimes attracted to roads.

The relatively low levels of habitat selection displayed by zebra indicate that they are less specialised in their resource requirements than the other two species. Zebra show strong second order selection that relates to their home range locations within the landscape, but within those home ranges, they utilise all habitats in proportion to their availability. Habitat generalists are more mobile and adaptable than habitat specialists, allowing compensatory movement in response to environmental change [63]. Wildebeest showed a stronger preference for tertiary floodplains than the other two species, which could cause vulnerability to environmental change. The Okavango Delta flood levels vary annually [64], and, between 2007–2012, there were several years with very high flood levels [65], restricting access to floodplains relied upon by grazers [30], and potentially preventing them from reaching additional resources on the other side of flooded channels. Changing vegetation and habitat composition within an ecosystem over several years can have strong effects on herbivores [66]. Relatively non-selective grazers such as buffalo altered their patterns of habitat selection in response to flooding levels in the Okavango Delta, moving away from floodplains and selecting more grassland and riparian woodland [30]. High flood levels in Lake Manyara, Tanzania, led to a population crash when wildebeest were unable to access floodplains on the lake edge and were stuck between water and riparian woodland, where they suffered high predation pressure from lion [67]. Higher flooding would lead to a higher ratio of secondary: tertiary floodplain, and most species avoided secondary floodplain, despite its role as a permanent water source. Higher water levels could further restrict herbivore movement and affect gene flow [68]. Loss of genetic connectivity can have severe impacts on a population, increasing vulnerability to disease and reducing their ability to adapt to changing environmental conditions [69].

The natural fluctuations of water flow in the Okavango Delta can lead to extreme changes in environmental conditions and resource availability from one year to the next [30], but also over longer time periods [65]. Species that are able to adapt rapidly to changing conditions are most likely to thrive in such an environment, whereas species with high habitat specificity and limited movement capacity are more likely to decline. High flood levels can cause some species to leave protected areas in search of adequate resources [30]. Buffalo, elephant and predators have all been expanding their ranges, moving closer to centres of human activity and into some areas outside of their usual range, in a similar way to moose in northern America [70]. Medium bodied herbivore species are more vulnerable to hunting and predation [71,72], so are likely to be naturally less bold than these larger species. Human population growth around the Okavango Delta in recent years could be restricting compensatory movement that may have happened during the previous period of high flooding, leading to the observed herbivore population declines.

## Conclusions

Larger home ranges sizes, lack of third order selection and a propensity to form larger, more fluid groups suggest that zebra are more adaptable and better able to track changing environmental conditions than tsessebe or wildebeest. The natural, stochastic environmental changes in flood levels in the Okavango Delta could have a detrimental impact on sedentary species such as tsessebe and wildebeest, especially during prolonged flooding periods lasting several years. In this ecosystem, collared tsessebe and wildebeest moved seasonally and changed their patterns of habitat selection, but they may have been constrained by their lower capacity for adaptive movement in response to changing conditions when compared to zebra. Climate change scenarios predict higher levels of environmental fluctuation, particularly in combination with proposed anthropogenic developments upstream of the Delta [73], so populations of sedentary herbivores may continue to decline. The floodplains of the Okavango Delta currently extend far beyond the boundaries of the protected and wildlife management areas, so expanding the protected areas, particularly during extended periods of high flooding, could provide more extensive areas for colonisation by vulnerable herbivore species. Buffer zones extending beyond protected areas could be established with flexible land use strategies, such that during periods of high flood, they could be used as additional eco-tourism areas, and during periods of low flood, they could be developed as cultural tourism destinations.

Rates of environmental change are likely to increase in the future, so only species that can adapt at a similar pace will survive [69]. High dispersal ability is key for species to track shifting resources [46], but they must also be able to move into optimal areas as resource distributions change. Many protected areas were established to conserve historical ecosystems [74], with little capacity for expansion [75]. Globally, protected areas have lower conservation value than randomly selected locations, thereby failing to address global conservation priorities [76]. Small-bodied species occurring at high densities benefit greatly from static protected areas, but species such as medium and large bodied herbivores may need more targeted conservation if their populations are to stabilise or even increase [77]. Buffer zones around existing protected areas could provide valuable space for species requiring access to limiting resources or habitats, as well as allowing mobile species to engage in adaptive movements [69].
